# Neutrophil-to-Lymphocyte Ratio Is Associated with Disease Progression in a Surveillance Cohort of Patients with Branch-Duct IPMN

**DOI:** 10.3390/jcm15197758

**Published:** 2026-10-08

**Authors:** Frederik J. Hansen, Peter Ewald, Luis Sorncharoen, Daniel R. Quast, Maximilian Stögbauer, Christian Torres Reyes, Renate Schlottmann, Christoph Zeidler, Mousa Ayoub, Bernadette Kaup, Anne Koch, Sarah Schellen, Lisa Weckheuer, Oscar Cahyadi, Alanna Ebigbo

**Affiliations:** Medizinische Klinik I, Gastroenterology, St. Josef–Hospital Bochum, Hospital of the Ruhr-University Bochum, Gudrunstraße 56, 44791 Bochum, Germany; peter.ewald@klinikum-bochum.de (P.E.); luis.sorncharoen@rub.de (L.S.); daniel.quast@klinikum-bochum.de (D.R.Q.); maximilian.stoegbauer@klinikum-bochum.de (M.S.); christian.torresreyes@klinikum-bochum.de (C.T.R.); renate.schlottmann@klinikum-bochum.de (R.S.); christoph.zeidler@klinikum-bochum.de (C.Z.); mousa.ayoub@klinikum-bochum.de (M.A.); bernadette.kaup@klinikum-bochum.de (B.K.); anne.koch@klinikum-bochum.de (A.K.); sarah.schellen@klinikum-bochum.de (S.S.); lisa.weckheuer@klinikum-bochum.de (L.W.); oscar.cahyadi@klinikum-bochum.de (O.C.); alanna.ebigbo@klinikum-bochum.de (A.E.)

**Keywords:** NLR, neutrophil-to-lymphocyte ratio, endoscopic ultrasound, EUS, IPMN, intraductal papillary mucinous neoplasms, branch-duct IPMN, BD-IPMN, worrisome feature, surveillance, biomarker

## Abstract

**Background**: A Branch-duct intraductal papillary mucinous neoplasms (BD-IPMNs) are common pancreatic precursor lesions requiring varying duration of surveillance because of their potential for malignant transformation. Current risk stratification relies predominantly on imaging-based criteria defined by the Kyoto guidelines, while reliable blood-based biomarkers are lacking. The neutrophil-to-lymphocyte ratio (NLR) has been associated with invasive IPMN in surgical resected cases, but its role in routine BD-IPMN surveillance has not been investigated. This study investigated the association between NLR and disease progression in a large surveillance cohort of patients with BD-IPMN. **Methods**: This retrospective two-center study included patients with image-based diagnosis of BD-IPMN who underwent at least one further follow-up endoscopic ultrasound (EUS) examination between 2006 and 2026 and had an available differential blood count. Disease progression was defined as the development of new worrisome features (WFs), high-risk stigmata (HRS), pancreatic resection for progressive disease, or IPMN-associated carcinoma. **Results**: A total of 285 patients undergoing 2207 EUS examinations were included, with a mean EUS surveillance duration of 4.8 years. During follow-up, 63 patients (22.1%) developed at least one WF, 4 (1.4%) developed HRS, 11 (3.9%) underwent pancreatic resection, and 2 (0.7%) developed IPMN-associated carcinoma. NLR was significantly elevated in patients who developed WFs (*p* = 0.0023), HRS (*p* < 0.0001), underwent pancreatic resection (*p* = 0.0006), or had high-grade dysplasia (*p* = 0.0002). Baseline continuous NLR showed no significant discriminatory ability for subsequent disease progression (AUC, 0.563; 95% CI, 0.482–0.635; *p* = 0.136). Patients with an NLR above the cohort median (>2.36) had approximately twofold higher odds of subsequent disease progression (OR, 2.02; 95% CI, 1.02–3.98). Longitudinal analyses demonstrated significant increases in NLR preceding the development of WFs (*p* < 0.0001), HRS (*p* = 0.0265), and pancreatic resection (*p* = 0.0272). Rising NLR was particularly associated with cyst growth, cyst size ≥30 mm, and main pancreatic duct dilation. **Conclusions**: Elevated and increasing NLR was associated with BD-IPMN progression and correlated with established markers of higher-risk disease. Longitudinal NLR assessment may represent a simple and widely available complementary biomarker for surveillance; however, its modest discriminatory performance warrants prospective validation before clinical implementation.

## 1. Introduction

The pancreatic ductal adenocarcinoma (PDAC) is one of the most lethal solid malignancies, with a 5-year overall survival rate of approximately 13% [1]. The incidence is rising at a rate of 0.5% to 1.0% per year, and PDAC is projected to become the second-leading cause of cancer-related mortality by 2030 [2]. The unfavorable prognosis of PDAC is largely driven by aggressive tumor biology and the asymptomatic nature of early disease, resulting in the majority of patients presenting with locally advanced or metastatic disease at diagnosis when curative surgical resection is no longer feasible [1,2]. Only 15–20% of patients present with resectable disease, underscoring the critical importance of identifying and monitoring precursor lesions before malignant transformation occurs [2,3].

Among the recognized precursors of PDAC, intraductal papillary mucinous neoplasms (IPMNs) represent the most common and clinically relevant entity. IPMNs are mucin-producing cystic neoplasms of the pancreatic ductal system, with a reported prevalence of 3–20% in the general population that increases with age [4]. The branch-duct subtype (BD-IPMN) is by far the most frequently encountered subtype, accounting for over 95% of IPMNs in clinical practice [5]. Although BD-IPMNs carry a lower risk of malignant transformation than main-duct IPMNs, the risk remains clinically relevant. Long-term surveillance studies have demonstrated cumulative incidences of high-grade dysplasia or invasive carcinoma of 3.3% at 5 years, 6.6% at 10 years, and up to 15% at 15 years [6]. Furthermore, patients with BD-IPMN have an approximately ninefold higher risk of developing pancreatic cancer than the age-matched general population [7]. With the increasing use of cross-sectional imaging, the incidental detection of BD-IPMNs has risen substantially, resulting in an increasing number of patients undergoing long-term surveillance [8,9]. Current management of BD-IPMN is guided by the 2024 revised International Association of Pancreatology (IAP) guidelines, known as the Kyoto guidelines [10,11]. These guidelines stratify patients according to the presence of worrisome features (WF) and high-risk stigmata (HRS). HRS, including obstructive jaundice, enhancing mural nodule ≥ 5 mm, main pancreatic duct (MPD) ≥ 10 mm, and suspicious or positive cytology, are considered indications for surgical resection [10,11]. WF such as cyst diameter ≥ 3 cm, MPD 5–9 mm, enhancing mural nodule < 5 mm, thickened cyst walls, abrupt change in MPD caliber with distal atrophy, lymphadenopathy, elevated CA 19-9, and cystic growth rate ≥ 2.5 mm/year, prompt further evaluation with endoscopic ultrasound and closer surveillance [10,12]. Despite these advances, the Kyoto guidelines have notable limitations. Their risk stratification relies predominantly on morphological imaging criteria, which are subject to interobserver variability and may not fully capture the biological behavior of individual lesions [13,14]. Validation studies have shown that the Kyoto model achieves only modest discrimination for high-grade dysplasia or invasive carcinoma, with reported AUC values as low as 0.62 in external cohorts [15]. Serum CA 19-9, the only routinely used serum biomarker incorporated in the guidelines, has limited sensitivity for early or low-volume disease [16,17]. Consequently, there is a recognized need for complementary, noninvasive, and readily accessible biomarkers that may improve risk stratification and inform surveillance strategies in patients with BD-IPMN [13,18].

The neutrophil-to-lymphocyte ratio (NLR) has emerged as one of the most extensively studied inflammation-based biomarkers in oncology. Derived from a routine complete blood count, NLR reflects the balance between pro-tumorigenic neutrophil-driven inflammation and anti-tumor lymphocyte-mediated immunity [19,20]. The biological rationale is well established: neutrophils promote tumor progression through angiogenesis, immune suppression, and extracellular matrix remodeling, while a relative lymphopenia reflects impaired adaptive anti-tumor immunity [21,22]. In the context of IPMN, several retrospective studies of surgically resected cohorts have demonstrated that elevated preoperative NLR is significantly associated with invasive carcinoma [23,24]. Additional studies have found that NLR can be used as a criterion to differentiate invasive from non-invasive IPMN [25,26]. However, the available studies have several important limitations, most notably their exclusive focus on surgically resected cohorts. This introduces an inherent selection bias toward higher-risk lesions, including main-duct or mixed-type IPMNs.

To date, no study has evaluated NLR in a dedicated surveillance cohort of patients with BD-IPMN. This study investigates whether NLR can serve as a dynamic biomarker of malignant progression and improve risk stratification during routine follow-up.

## 2. Materials and Methods

### 2.1. The Study Design

**Figure 1:**
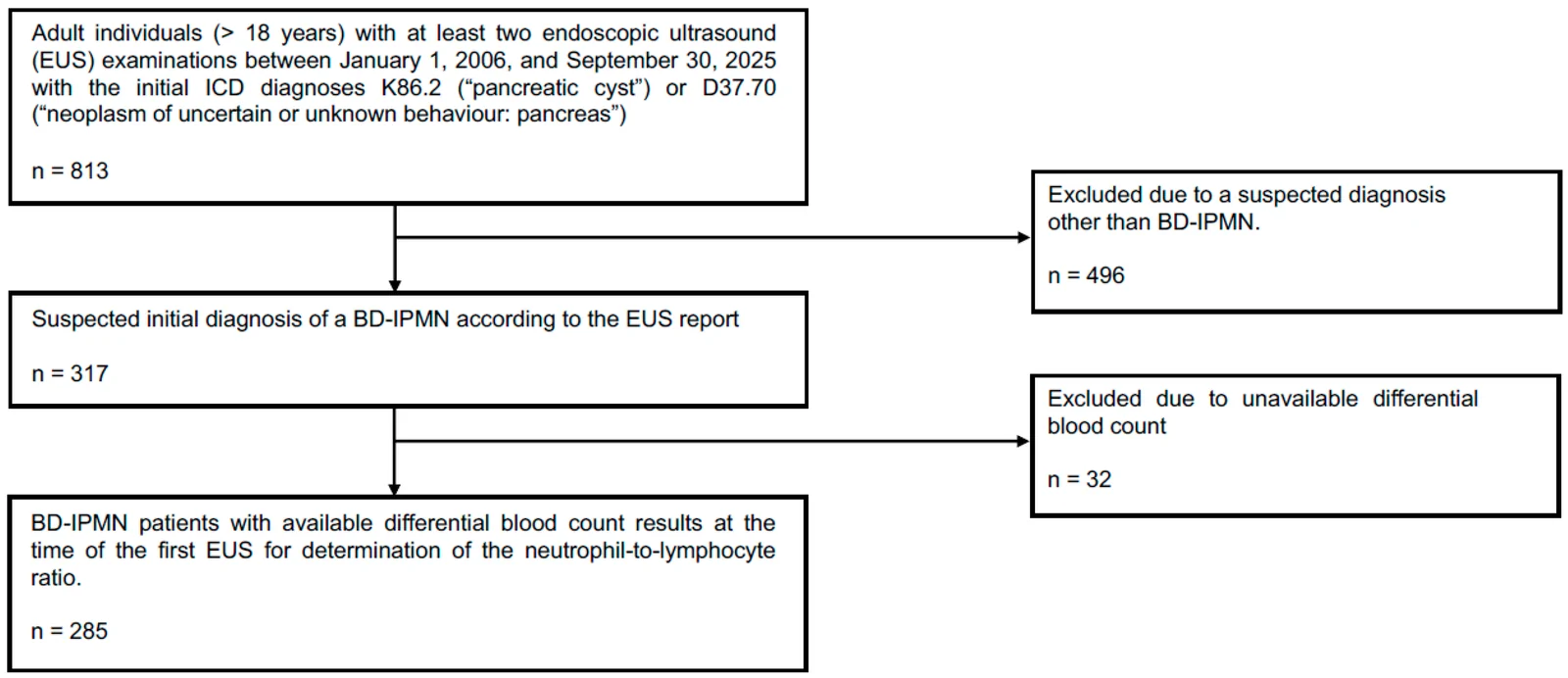
Selection of study patients.

The present study was conducted as a retrospective two-center analysis. Patients who underwent at least two endoscopic ultrasound (EUS) examinations of the pancreas at St. Josef Hospital Bochum, Ruhr University Bochum, and Martin-Luther Hospital Wattenscheid between 1 January 2006, and 30 September 2025, and who had an ICD-coded diagnosis of K86.2 (“pancreatic cyst”) or D37.70 (“neoplasm of uncertain or unknown behavior of the pancreas”) were initially screened for inclusion. Data extraction was performed using the ORBIS© clinical information system. Neutrophil and lymphocyte counts were obtained from automated differential blood count analyses of EDTA blood samples. These measurements were performed as part of routine clinical care prior to EUS and were not specifically collected for the purposes of this study. Subsequently, patients with suspected branch-duct intraductal papillary mucinous neoplasm (BD-IPMN) at the initial EUS examination were selected for further analysis. Patients were included in the final study cohort if a differential blood count obtained at or closest to the time of the initial EUS examination was available. A flowchart illustrating the study design and patient selection process is provided in Figure 1. The criteria used to define worrisome features (WF) and high-risk stigmata (HRS) were based on the revised Kyoto guidelines and are outlined in the Introduction [10]. In the subsequent analysis, we evaluated the risk of progression of BD-IPMNs. Progression was defined as the development of new WF, HRS, pancreatic resection, or the development of malignancy.

Exclusion criteria included age < 18 years, the presence of invasive pancreatic carcinoma at the time of the initial EUS examination, main-duct or mixed-type IPMN, an alternative initial diagnosis of a pancreatic cystic lesion, fewer than two EUS examinations performed at the study center, absence of a differential blood count, and incomplete clinical or imaging data in the ORBIS© (Trier, Germany) clinical information system.

### 2.2. Statistical Analysis

Statistical analyses were performed using GraphPad Prism© (Version 11.0.2, La Jolla, CA, USA). Continuous variables were expressed as mean ± standard deviation (SD) and compared using either unpaired or paired *t*-tests, as appropriate. Unpaired *t*-tests were applied for comparisons between independent groups, whereas paired *t*-tests were used for comparisons of repeated measurements within the same patients.

For OR analysis, patients were stratified into low- and high-NLR groups according to the cohort median of 2.36. The odds ratio (OR) with corresponding 95% confidence interval (95% CI) was calculated to assess the unadjusted association between NLR category and subsequent disease progression. Due to the retrospective study design and insufficiently complete data on relevant potential confounders, no multivariable adjustment was performed.

Receiver operating characteristic (ROC) curve analysis was performed to assess the discriminatory ability of baseline NLR for subsequent disease progression. The area under the ROC curve (AUC) with the corresponding 95% confidence interval (CI) was calculated. All statistical tests were two-tailed, and *p* values < 0.05 were considered statistically significant.

## 3. Results

### 3.1. Baseline Characteristics

Overall, 285 patients were included in this retrospective analysis (Figure 1). The mean age of the study population was 66.9 years, with a predominance of female patients (65.6%). The mean surveillance duration was 4.8 years, with an average of 7.7 EUS examinations per patient. In total, 2207 EUS examinations were performed and analyzed. The mean neutrophil-to-lymphocyte ratio at the initiation of surveillance was 2.6. The majority of BD-IPMNs were multifocal (37.9%), followed by the pancreatic body (35.8%), head (15.8%), and tail (10.5%). Approximately half of the patients (53.7%) had a single lesion, whereas 20.0% had four or more pancreatic lesions. The mean size of the largest BD-IPMN at baseline was 11.6 mm. During the surveillance period, 63 patients (22.1%) developed at least one worrisome feature (WF) according to the Kyoto guidelines, whereas only 4 patients (1.4%) developed high-risk stigmata (HRS). Pancreatic resection for BD-IPMN was performed in 11 patients (3.9%), and 2 patients (0.7%) developed IPMN-associated carcinoma. The clinical characteristics of the study population are summarized in Table 1.

### 3.2. Neutrophil-to-Lymphocyte Ratio and Development of Worrisome Features in BD-IPMN Patients

**Figure 2:**
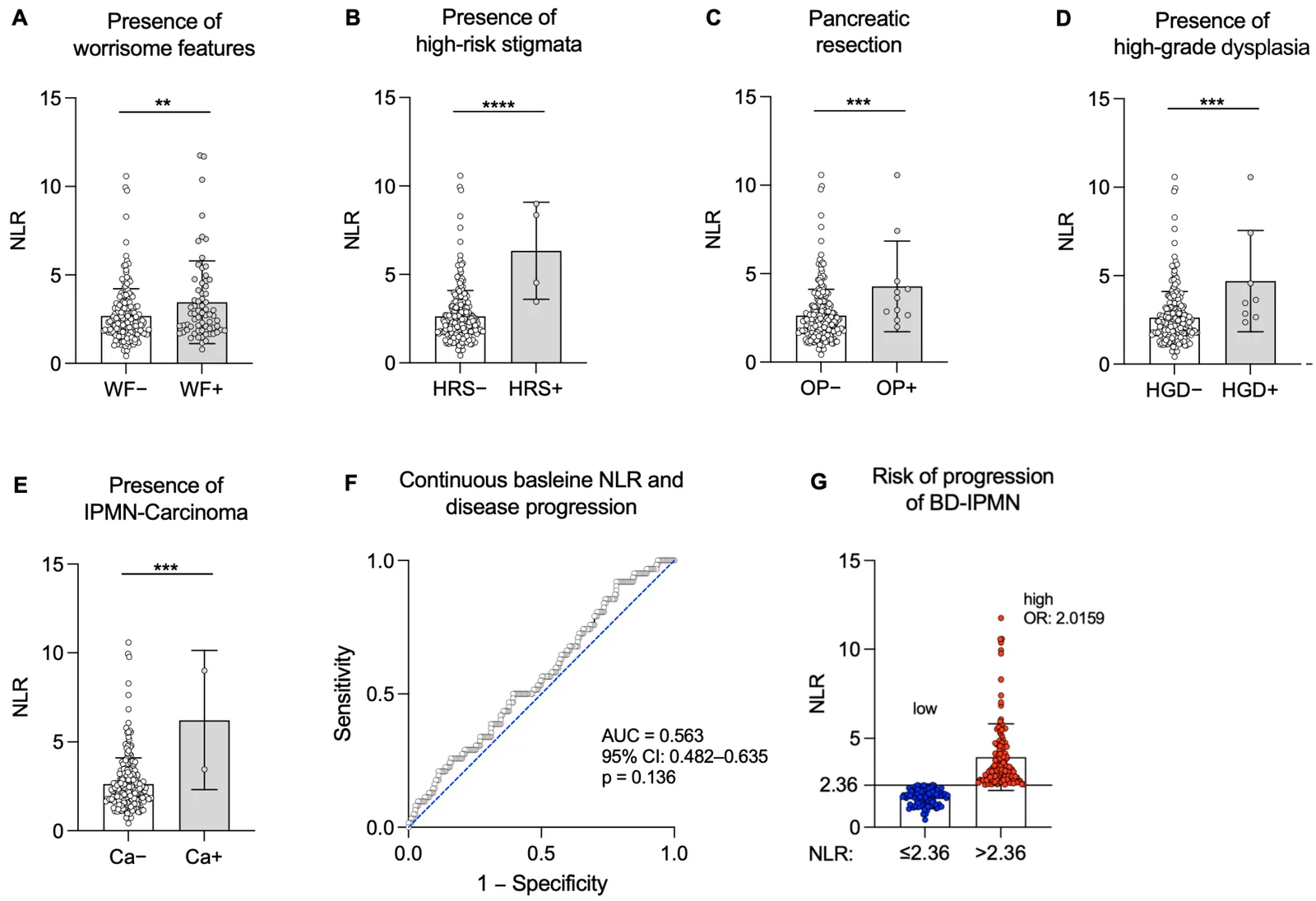
Elevated neutrophil to lymphocyte ratio (NLR) correlated with progressive BD-IPMNs. NLR in patients with the absence of worrisome features (WF−) compared to patients with new diagnosed worrisome features (WF+) (**A**); NLR in patients with the absence of high-risk stigmata (HRS-) compared to patients with new diagnosed high-risk stigmata (HRS+) (**B**); NLR in patients without performed pancreatic resection (OP−) compared with patients immediately prior to required pancreatic resection (OP+) (**C**); Comparison of NLR in patients whose pancreatic lesion was not treated by surgery or high-grade dysplasia (HGD-) versus patients with high-grade histology (HGD+). (**D**); NLR in patients without clinical evidence of pancreatic carcinoma (Ca−) compared with patients with newly diagnosed IPMN-associated carcinoma (Ca+) (**E**); receiver operating characterstics (ROC) curve demonstrating the discriminatory performance of continuous baseline NLR for disease progression (progression was defined as the development of at least one WFs, one HRS, pancreatic resection, or the development of carcinoma) (**F**); NLR grouped according the median 2.36 in all BD-IPMN patients defining the risk of progression of BD-IPMN (**G**); **** = *p* < 0.0001; *** = *p* < 0.001; ** = *p* < 0.01.

All patients who developed a new WF had a significantly higher NLR than patients who did not develop any WF (*p* = 0.0023) (Figure 2A). The specific types of WFs observed are summarized in Table 2. Similarly, patients who developed HRS (*n* = 4) had a significantly higher NLR than those who did not develop HRS (*n* = 281) (*p* < 0.0001) (Figure 2B). The individual HRS findings are also detailed in Table 2.

Patients who underwent surgery for progressive BD-IPMN (*n* = 11) demonstrated a significantly elevated NLR compared with those who did not undergo resection (*p* = 0.0006) (Figure 2C). Histopathological analyses were performed by comparing patients with high-grade dysplasia (HGD) or invasive carcinoma (*n* = 7) to patients who either did not undergo surgery or had histological findings of no dysplasia or low-grade dysplasia (*n* = 277). Patients with HGD or invasive carcinoma showed significantly higher preoperative NLR values than the comparison group (*p* = 0.0002) (Figure 2D). In a subgroup analysis of IPMN-associated carcinomas, one patient underwent surgical resection, while the second developed metastatic disease and was diagnosed histologically by EUS-guided fine-needle biopsy without surgery.

Both patients with IPMN-associated carcinoma had elevated pre-interventional NLR values compared with patients without evidence of carcinoma (*p* = 0.0008) (Figure 2E). Detailed histopathological findings of the 11 resected lesions of the patients are provided in Table 2.

Finally, we examined whether elevated NLR levels were associated with an increased likelihood of disease progression. Progression was defined as the development of a new WF, HRS, pancreatic resection for progressive disease, or the diagnosis of IPMN-associated carcinoma. ROC curve analysis of the baseline continuous NLR showed no significant discriminatory ability of baseline NLR for subsequent disease progression (AUC, 0.5630; 95% CI, 0.4823–0.6347; *p* = 0.1356) (Figure 2F). To further evaluate the discriminatory performance of the NLR classification used for the risk analysis, patients were subsequently stratified into low- and high-NLR groups according to the cohort median of 2.36. Patients with an NLR > 2.36 had approximately twofold higher odds of subsequent disease progression compared with patients with an NLR ≤ 2.36 (OR, 2.0159; 95% CI, 1.0207–3.9812) (Figure 2G).

### 3.3. NLR Increased over Time in Patients with Progressive BD-IPMN

**Figure 3:**
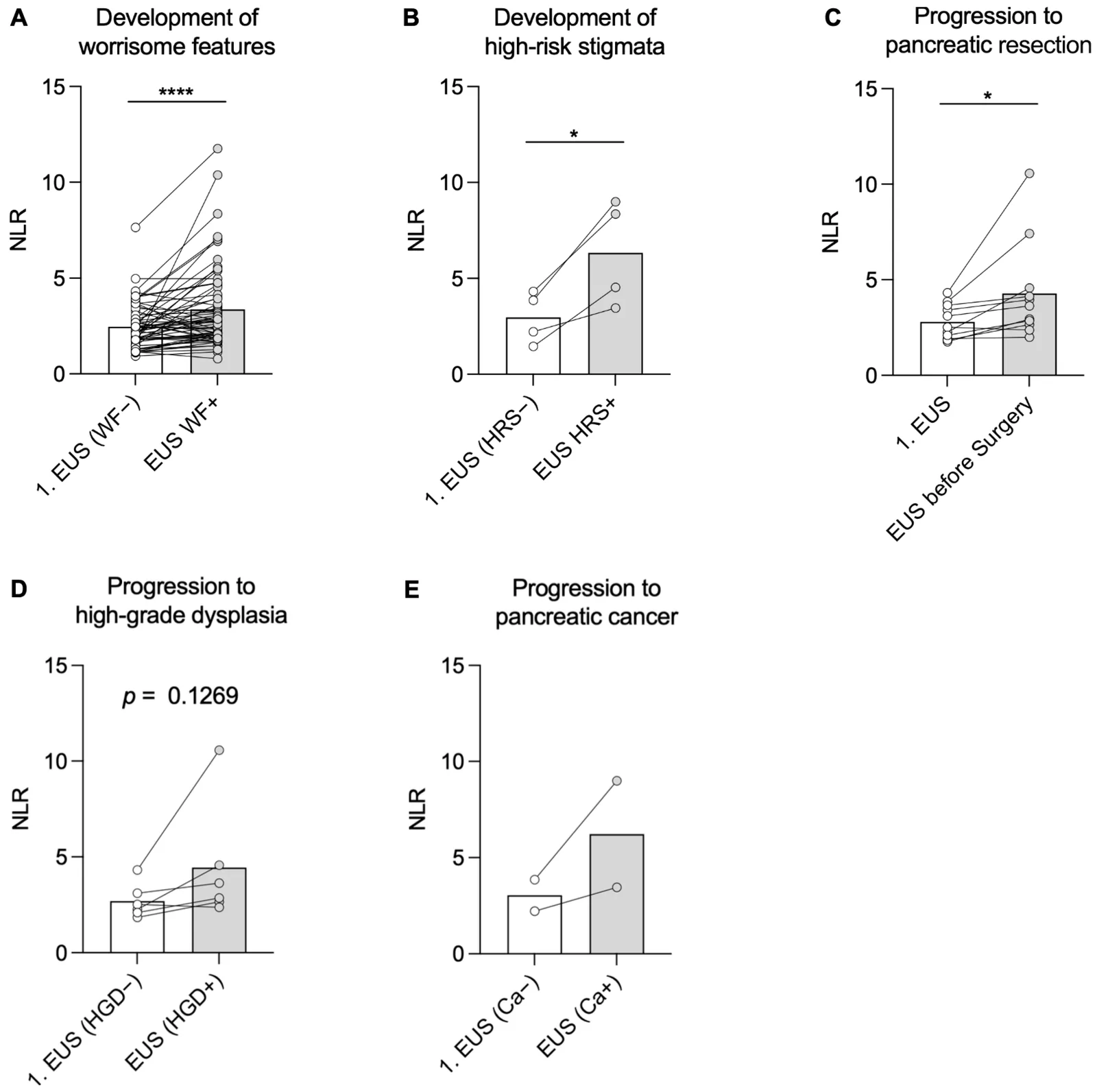
Neutrophil-to-lymphocyte ratio (NLR) as an individual longitudinal biomarker for the progression of BD-IPMN. NLR in patients who developed worrisome features during follow-up: comparison between the initial EUS without worrisome features (WF−) and the first detection of a newly developed worrisome feature (WF+) (**A**); NLR in patients who developed high-risk stigmata during follow-up: comparison between the initial EUS without high-risk stigmata (HRS−) and the first detection of a newly developed high-risk stigmata (HRS+) (**B**); Longitudinal changes in NLR in patients requiring pancreatic resection during follow-up (**C**); NLR over the course of follow-up in patients with subsequent development of high-grade dysplasia (HGD+) (**D**) and IPMN-associated carcinoma (**E**); **** = *p* < 0.0001; * = *p* < 0.05.

Patients who developed at least one new WF during follow-up (*n* = 63) showed a significant increase in NLR between the start of surveillance (WF−) and at the time of first WF detection (WF+) (*p* < 0.0001) (Figure 3A). Similarly, among patients who developed HRS, NLR values were significantly higher at the time of HRS detection than at baseline (*p* = 0.0265) (Figure 3B). Patients who subsequently underwent pancreatic resection showed a significantly lower NLR at the start of surveillance compared with the NLR immediately prior to surgery (*p* = 0.0272) (Figure 3C). Regarding the development of high-grade dysplasia (HGD), there was a trend toward increased NLR, although this did not reach statistical significance (*p* = 0.1269) (Figure 3D). For patients who developed pancreatic carcinoma at the site of the IPMN, NLR values showed a non-significant increase from baseline to the time of carcinoma diagnosis (*p* = 0.3488) (Figure 3E).

### 3.4. NLR and Specific Worrisome Features in BD-IPMN

**Figure 4:**
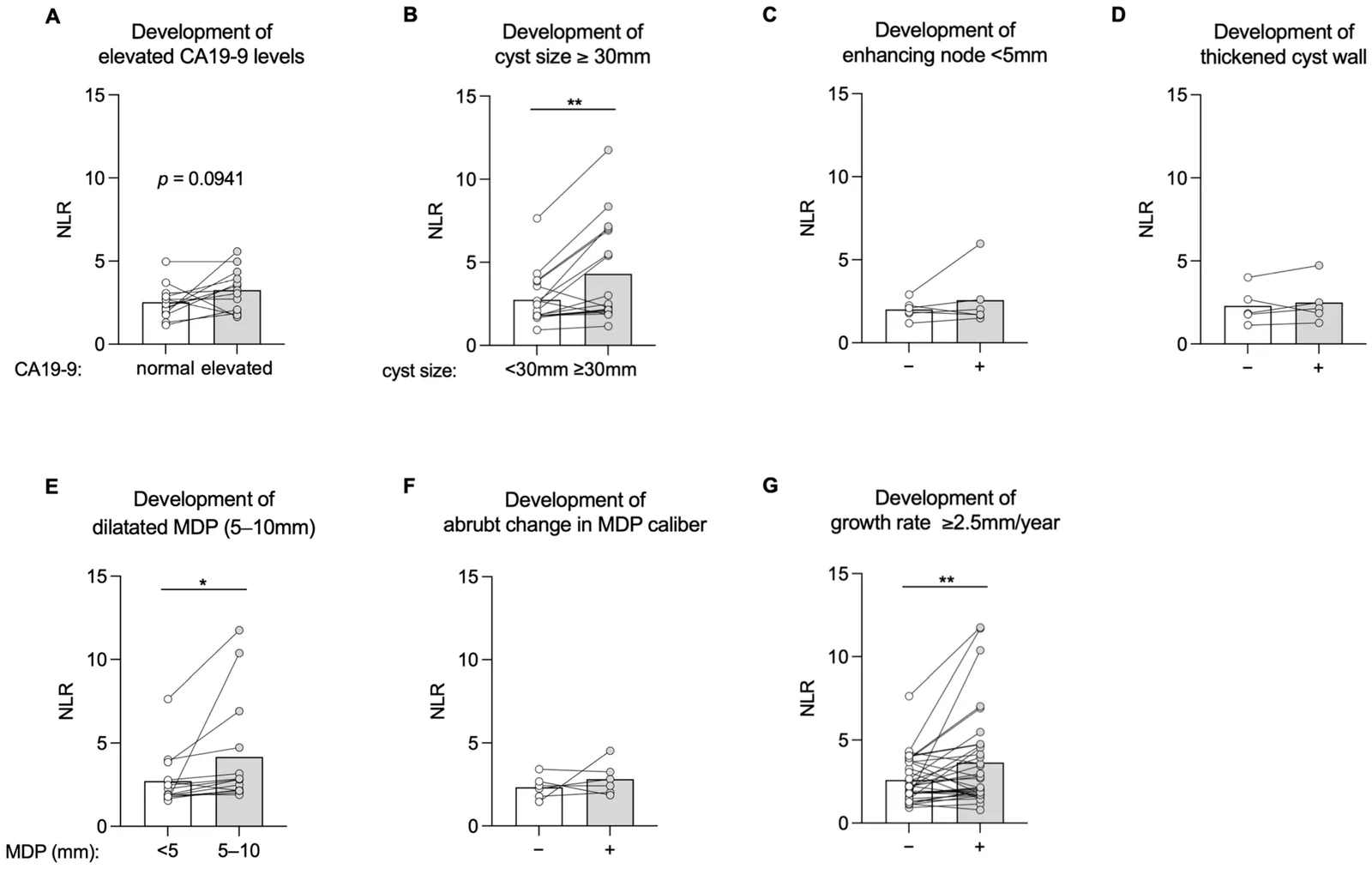
Longitudinal changes in NLR according to worrisome features. NLR in patients who developed worrisome features during follow-up. Elevated CA19-9 levels (**A**), cyst size ≥ 30 mm (**B**), enhancing node < 5 mm (**C**), thickened cyst wall (**D**), dilated main pancreatic duct (MDP) 5–10 mm (**E**), abrupt change in MDP caliber with distal atrophy (**F**), growth rate of ≥2.5 mm/year (**G**); ** = *p* < 0.01; * = *p* < 0.05.

We next examined whether the observed increase in NLR was consistent across individual WFs or differed according to the specific WF. Patients who developed elevated CA19-9 levels during follow-up showed a non-significant increase in NLR (*p* = 0.0941) (Figure 4A). In contrast, patients whose cysts exceeded 30 mm showed a significant increase in NLR (*p* = 0.0033) (Figure 4B). No clear trend in NLR was observed for patients developing an enhancing mural nodule < 5 mm (*p* = 0.3325) (Figure 4C) or a thickened cyst wall (*p* = 0.4951) (Figure 4D), although the number of patients in these subgroups was small. Patients who developed a main pancreatic duct (MPD) dilatation showed a significant increase in NLR (*p* = 0.0467) (Figure 4E). In patients with an abrupt change in MPD caliber accompanied by distal atrophy, no significant difference in NLR was observed (*p* = 0.4275) (Figure 4F). However, patients with a cyst growth rate of ≥2.5 mm per year demonstrated a significant increase in NLR (*p* = 0.0085) (Figure 4G).

## 4. Discussion

In this EUS-based surveillance cohort of patients with BD-IPMN, elevated NLR was significantly associated with disease progression, including the development of worrisome features (WF), high-risk stigmata (HRS), the need for pancreatic resection, and advanced histopathology. Moreover, NLR increased longitudinally in patients with progressive disease and was particularly associated with the development of cyst growth, cyst size ≥ 30 mm, and main pancreatic duct dilation. Patients with an NLR above the cohort median had a twofold increased risk of harboring progressive BD-IPMN, highlighting the potential value of NLR as a readily available biomarker for risk stratification during surveillance.

Importantly, the discriminatory performance of baseline NLR should be interpreted in the context of its modest magnitude. When analyzed as a continuous variable, baseline NLR showed limited and non-significant discrimination for subsequent disease progression in our analysis. Thus, although an NLR > 2.36 was associated with an approximately twofold increased risk of disease progression, the non-significant AUC indicates that NLR should not be regarded as a standalone predictive or diagnostic marker. Rather, our findings support its potential role as a complementary biomarker that may provide additional biological information alongside established imaging and clinical parameters. Prospective studies incorporating NLR into multivariable prediction models are required to determine whether it provides incremental prognostic value beyond established risk factors.

**Figure 5:**
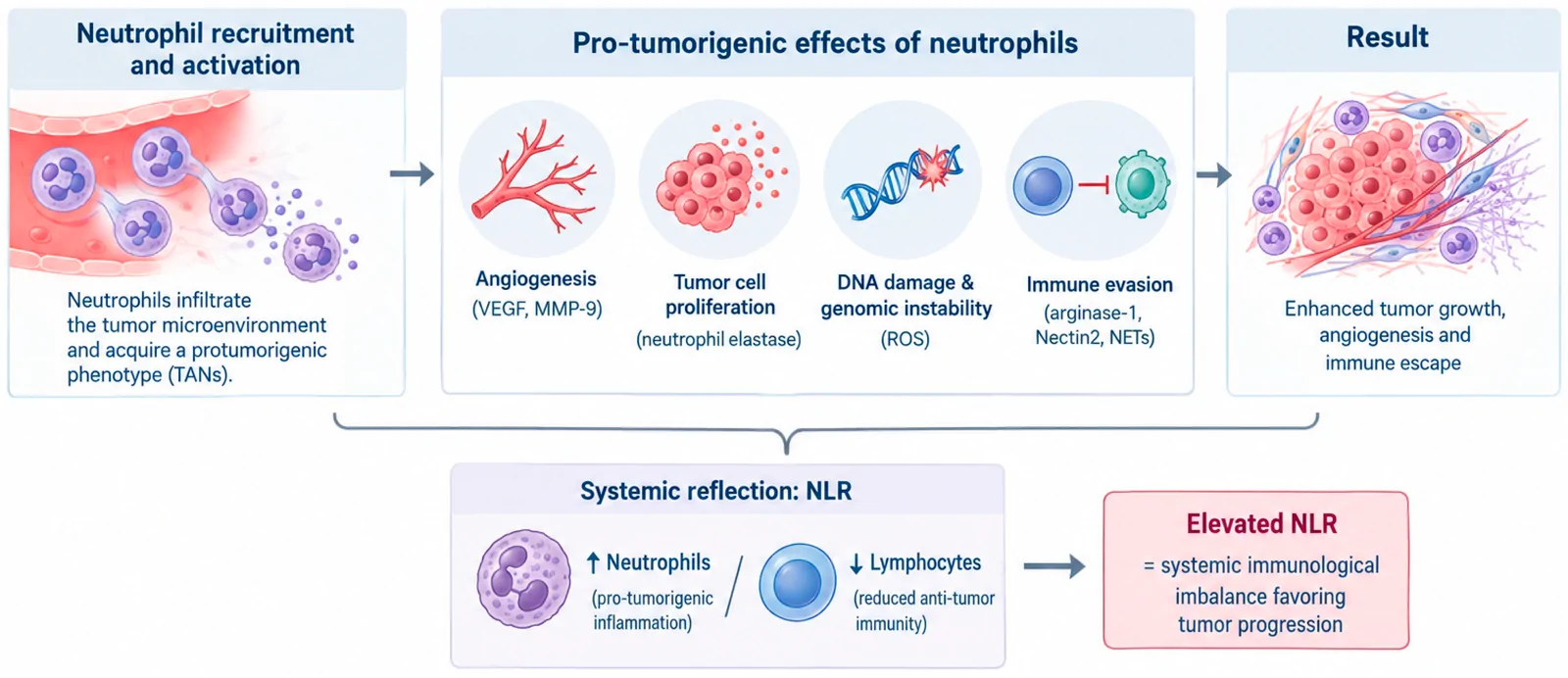
A schematic overview of the mechanisms through which neutrophils promote tumour progression. Figure was created with the assistance of OpenAI’s GPT-5.6 Luna.

Neutrophils are the most abundant circulating leukocytes and have traditionally been regarded as short-lived effectors of innate immunity. However, it is now well established that neutrophils recruited into the tumor microenvironment, termed tumor-associated neutrophils (TANs), undergo functional reprogramming and can adopt a protumorigenic phenotype (N2) that actively promotes cancer development and progression [27,28,29]. In the context of pancreatic neoplasia specifically, TANs have been shown to facilitate tumor progression through several interconnected mechanisms: secretion of vascular endothelial growth factor (VEGF) and matrix metalloproteinase-9 (MMP-9), which drive angiogenesis and extracellular matrix remodeling; release of neutrophil elastase, which directly stimulates tumor cell proliferation; production of reactive oxygen species (ROS) that induce DNA damage and genomic instability; and suppression of cytotoxic CD8+ T-cell responses through arginase-1 secretion and upregulation of immune checkpoint molecules such as Nectin2 [30,31,32]. Furthermore, neutrophil extracellular traps (NETs), web-like chromatin structures released by activated neutrophils, have been shown to be substantially enriched within the PDAC tumor microenvironment, where they promote angiogenesis, stromal remodeling, and immune evasion [33,34]. A schematic overview of the underlying pathogenesis is shown in Figure 5.

Critically, these protumorigenic mechanisms involving neutrophils are not restricted to established invasive carcinoma but may already be active during the early stages of pancreatic neoplastic transformation. Myeloid cell infiltration, including neutrophils and myeloid-derived suppressor cells, has been observed surrounding early PanIN-I lesions, suggesting that an inflammatory microenvironment may develop well before the onset of invasive disease [32].

The NLR, as a peripheral blood marker, is thought to reflect this tumor-immune dynamic at the systemic level. The neutrophil count serves as a surrogate for the protumorigenic, proangiogenic, and immunosuppressive inflammatory response, while the lymphocyte count reflects the adaptive antitumor immune surveillance capacity [35]. An elevated NLR therefore captures a systemic immunological imbalance that favors tumor promotion over tumor suppression. This interpretation is supported by the observation in the present study that NLR increased longitudinally in patients with progressive disease, mirroring the temporal evolution of the local inflammatory microenvironment during the dysplasia-carcinoma sequence. Sugimachi et al. similarly reported that NLR was significantly higher in patients with high-grade or invasive IPMN during the year preceding surgery, suggesting that changes in NLR may accompany disease progression and could potentially provide a dynamic marker during surveillance [36]. The associations observed in the present study between elevated NLR and the development of cyst growth, cyst size ≥30 mm, and main pancreatic duct dilation are biologically plausible and align with current concepts of inflammation-driven pancreatic tumorigenesis. Neutrophil-derived mediators, including VEGF and MMP-9, promote angiogenesis, vascular permeability, and extracellular matrix remodeling, processes that may facilitate cyst expansion and progressive structural changes within the pancreatic parenchyma, including fibrosis, ductal remodeling, and alterations of the cyst wall and epithelial architecture.

Concurrently, the inflammatory microenvironment shaped by tumor-associated neutrophils and neutrophil extracellular traps can alter ductal epithelial homeostasis, promote mucin production, and contribute to ductal remodeling and dilation [31,33]. Importantly, these morphological alterations constitute established WF according to the Kyoto guidelines. Our findings therefore suggest that NLR may reflect the biological processes driving disease progression rather than simply serving as a marker of its radiographic manifestations.

In this context, NLR may represent a systemic surrogate marker of the inflammatory and immunological changes that precede the emergence of guideline-defined risk features. These observations may have important implications for clinical practice. Current surveillance strategies for BD-IPMN are largely driven by structural changes detected on imaging, with the appearance of WF frequently triggering intensified surveillance and invasive diagnostic procedures such as EUS. The present data suggest that longitudinal assessment of NLR could complement existing imaging-based approaches by providing a readily available measure of disease biology. In particular, a persistently increasing NLR may identify patients at heightened risk of progression before overt morphological changes become apparent. Such an approach would acknowledge that biological progression may precede radiographic progression and could enable earlier identification of high-risk lesions. Conversely, stable NLR values over prolonged follow-up may indicate biological stability and help identify patients at lower risk of progression. If validated in prospective studies, incorporation of NLR into surveillance algorithms could refine patient selection for EUS and other invasive diagnostic procedures, thereby improving individualized risk stratification while potentially reducing unnecessary interventions in selected low-risk patients.

While NLR is simple, inexpensive, universally available, and reproducible, making it an attractive candidate for integration into clinical risk stratification models [20,37], several inherent limitations must be acknowledged when interpreting its clinical significance in the context of BD-IPMN surveillance. First, NLR is a nonspecific marker of systemic inflammation and is influenced by a wide range of physiological, demographic, and environmental factors independent of the underlying pancreatic pathology. A large population-based analysis of 48,023 adults from the National Health and Nutrition Examination Survey demonstrated that sex, age, race/ethnicity, body mass index, marital status, physical activity, smoking history, and alcohol consumption are all independently associated with NLR magnitude [38]. In particular, smoking has a well-documented dose-dependent effect on NLR: cigarette smoking disproportionately increases neutrophil counts relative to lymphocytes, and NLR rises in a linear fashion with cumulative pack-years [39]. Importantly, smoking cessation has been shown to significantly reduce NLR in tandem with other inflammatory and oxidative stress markers [40]. Given that smoking is also an established risk factor for pancreatic malignancy, this represents a potential confounding pathway that is difficult to fully disentangle. Owing to the retrospective nature of the study, data on patients’ smoking status and the timing of their last cigarette prior to blood collection were not available.

Second, NLR is acutely elevated by intercurrent infections, sepsis, and any systemic inflammatory condition, all of which may be present incidentally at the time of routine blood sampling during surveillance [41,42]. Corticosteroid use, whether exogenous or endogenous, markedly alters NLR by simultaneously increasing neutrophil release from the bone marrow and marginated pool while inducing lymphocyte apoptosis and redistribution; even a single dose of moderate-dose corticosteroids can double the NLR within 24 h [43,44]. Furthermore, chronic comorbidities prevalent in the BD-IPMN surveillance population, including obesity, diabetes mellitus, cardiovascular disease, and chronic obstructive pulmonary disease, are each independently associated with elevated NLR through distinct inflammatory mechanisms [45,46,47].

Third, NLR shows significant circadian variation. Neutrophil counts in peripheral blood peak during the late afternoon, while lymphocytes reach their highest levels around midnight, driven by rhythmic cortisol and epinephrine release [48,49,50]. This diurnal fluctuation means that the timing of blood sampling can meaningfully influence NLR values, a variable that is rarely standardized in retrospective studies or routine clinical practice.

Further limitations should be considered as well. The relatively small patient cohort limits statistical power, particularly for analyses of NLR in specific WF, HRS, or histopathological subgroups. Additionally, the observed associations between NLR and disease progression were unadjusted. Due to the retrospective nature of the study and the lack of sufficiently complete information on relevant potential confounders, including smoking status, alcohol consumption, comorbidities, and medication use, multivariable analysis was not performed. Therefore, an independent association between NLR and disease progression cannot be established, and residual confounding cannot be excluded. Furthermore, the retrospective design introduces variability in surveillance intervals, clinical decision-making, and timing of laboratory assessments. Moreover, the development of a new WF does not necessarily reflect true malignant progression, especially given the very low number of patients who developed carcinoma. One possible explanation for these findings is the unusually high frequency of EUS surveillance in our cohort, with patients undergoing examinations on average every 7.5 months, even in the absence of WFs or HRS. Notably, the cohort also showed a high incidence of WFs, with 22.1% of patients developing at least one during follow-up. Given that only two cases of carcinoma occurred in the entire cohort, these data raise the question of whether the current definition of WFs should be reconsidered, or whether certain WFs should be weighted differently in risk assessment.

Consequently, while elevated NLR appears associated with disease progression, prospective studies are needed to validate its role in risk stratification and clinical management.

## 5. Conclusions

In this large EUS surveillance cohort of patients with BD-IPMN, elevated NLR was significantly associated with disease progression, including the development of worrisome features, high-risk stigmata, the treatment-indication of pancreatic resection, and advanced histopathology. Moreover, NLR increased over time in patients with progressive disease, suggesting that longitudinal NLR assessment may provide complementary information on disease activity during BD-IPMN surveillance.

## Figures and Tables

**Table 1:** Characteristics of study patients.

Variable	BD-IPMN Patients (*n* = 285)
Age (in years), mean (SD)	66.9 (±10.4)
Sex	
Female	187 (65.6)
Male	98 (34.4)
Observation time (in years), mean (SD)	4.8 (±3.4)
Total number of EUS	2207
Number of EUS/patient, mean (SD)	7.7 (±5.9)
Initial Neutrophil-to-lymphocyte ratio	2.6 (±1.5)
Localisation of the BD-IPMN	
Head	45 (15.8)
Body	102 (35.8)
Tail	30 (10.5)
Multilocular	108 (37.9)
Initial number of lesions	
1	153 (53.7)
2	43 (15.1)
3	32 (11.2)
≥4	57 (20.0)
Initial size of the largest BD-IPMN (in mm), mean (SD)	11.6 (±6.2)
Development of at least one worrisome feature	63 (22.1)
Development of high-risk stigmata	4 (1.4)
Pancreatectomy for IPMN	11 (3.9)
Development of IPMN-associated carcinoma	2 (0.7)

Data are absolute samples and percentages unless otherwise specified. SD: standard deviation.

**Table 2:** Detailed characterization of patients with progression of BD-IPMN.

New Developed Worrisome Features	*n* = 98
Pancreatitis	2 (2.0)
Elevated CA19-9 levels	13 (13.3)
Cyst size ≥ 30 mm	17 (17.3)
Enhancing mural nodule < 5 mm	6 (6.1)
Thickened/enhancing cyst wall	5 (5.1)
MDP ≥ 5 mm and <10 mm	14 (14.3)
Abrupt change in caliber of MPD with distal atrophy	6 (6.1)
Cystic growth rate ≥ 2.5 mm	35 (35.7)
New developed high-risk stigmata	*n* = 4
Enhancing mural nodule ≥ 5 mm or solid component	2 (50.0)
MDP ≥ 10 mm	1 (25.0)
Suspicious or positive results of cytology	1 (25.0)
Pancreatectomy for IPMN	*n* = 11
Adenocarcinoma	1 (9.1)
IPMN (gastric type) with no dysplasia	1 (9.1)
IPMN (gastric type) with low-grade dysplasia	1 (9.1)
IPMN (gastric type) with high-grade dysplasia	3 (27.3)
IPMN adenoma with high-grade dysplasia	2 (18.2)
Serous cystic neoplasm (SCN)	1 (9.1)
Intraductal oncocytic papillary neoplasm (IOPN) with high-grade dysplasia	1 (9.1)
Unremarkable	1 (9.1)

Data are absolute samples and percentages unless otherwise specified.

## Data Availability

For all data requests, please contact the corresponding author.
